# *Navicula* sp. Sulfated Polysaccharide Gels Induced by Fe(III): Rheology and Microstructure

**DOI:** 10.3390/ijms17081238

**Published:** 2016-07-30

**Authors:** Diana Fimbres-Olivarría, José Antonio López-Elías, Elizabeth Carvajal-Millán, Jorge Alberto Márquez-Escalante, Luis Rafael Martínez-Córdova, Anselmo Miranda-Baeza, Fernando Enríquez-Ocaña, José Eduardo Valdéz-Holguín, Francisco Brown-Bojórquez

**Affiliations:** 1 DICTUS, Department of Scientific and Technological Investigations, University of Sonora, Hermosillo, Sonora 83000, Mexico; diana.fimbreso@a2004.uson.mx (D.F.-O.); jalopez@guayacan.uson.mx (J.A.L.-E.); lmtz@guayacan.uson.mx (L.R.M.-C.); fenrquez@guayacan.uson.mx (F.E.-O.); jvaldez@guayacan.uson.mx (J.E.V.-H.); 2 CIAD, A.C., Research Center for Food and Development, Hermosillo, Sonora 83000, Mexico; jmarquez@estudiantes.ciad.mx; 3 UES, State University of Sonora, Navojoa, Sonora 85875, Mexico; anselmo.miranda@ues.mx; 4 Department of Polymers and Materials, University of Sonora, Hermosillo, Sonora 83000, Mexico; fbrown@guaymas.uson.mx

**Keywords:** *Navicula* sp., sulfated polysaccharide, gelation, trivalent ions

## Abstract

A sulfated polysaccharide extracted from *Navicula* sp. presented a yield of 4.4 (% *w*/*w* dry biomass basis). Analysis of the polysaccharide using gas chromatography showed that this polysaccharide contained glucose (29%), galactose (21%), rhamnose (10%), xylose (5%) and mannose (4%). This polysaccharide presented an average molecular weight of 107 kDa. Scanning electron microscopy (SEM) micrographs showed that the lyophilized *Navicula* sp. polysaccharide is an amorphous solid with particles of irregular shapes and sharp angles. The polysaccharide at 1% (*w*/*v*) solution in water formed gels in the presence of 0.4% (*w*/*v*) FeCl_3_, showing elastic and viscous moduli of 1 and 0.7 Pa, respectively. SEM analysis performed on the lyophilized gel showed a compact pore structure, with a pore size of approximately 150 nm. Very few studies on the gelation of sulfated polysaccharides using trivalent ions exist in the literature, and, to the best of our knowledge, this study is the first to describe the gelation of sulfated polysaccharides extracted from *Navicula* sp.

## 1. Introduction

For several years, marine microalgae have been of great interest because they contain a great variety of bioactive compounds with biotechnological potential, especially in the biomedical, pharmaceutical, nutraceutical, and cosmetic areas. Among the wide variety of microalgae used for biotechnological purposes are the diatoms, whose principal purpose is the production of biodiesel due to their high lipid content [1]. Some diatoms are benthic microalgae; they produce mucilage that binds them to their substrate. This mucilage is a matrix with a high content of extracellular polymeric substances, including polysaccharides [2]. The marine microalgae of the *Navicula* genus are benthic diatoms, and several bioactive compounds of commercial interest can be obtained from them, including polysaccharides [3,4,5]. Several studies have proven that microalgae polysaccharides have great potential as antiviral, antibacterial, and antioxidant compounds, among other uses. Despite some research on their applications appearing already, information on sulfated polysaccharides from species of the genus *Navicula* is still scarce. Currently, no reports exist on the gelation behavior of sulfated polysaccharides from this genus. However, there is some evidence that sulfate polysaccharides can form gels in the presence of trivalent ions, as shown for λ-carrageenan from seaweeds [6]. The aim of this study was to investigate the gelation of a sulfated polysaccharide from *Navicula* sp. in the presence of trivalent iron ions and to study the rheological and microstructural characteristics of the gel formed.

## 2. Results and Discussions

### 2.1. Polysaccharide Characteristics

**Figure 1:**
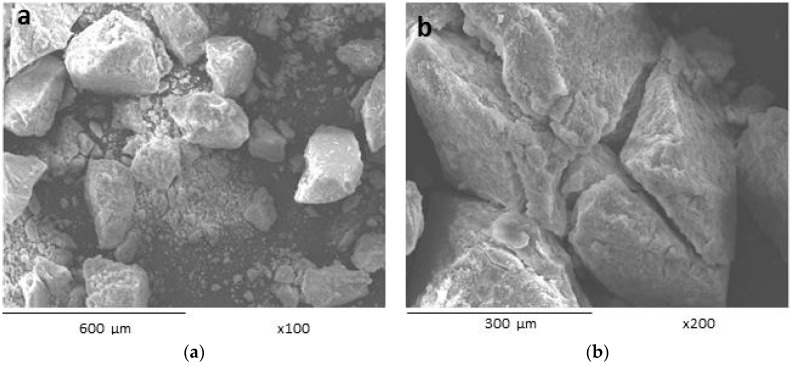
Scanning electron microscopy (SEM) micrographs of lyophilized polysaccharide extracted from *Navicula* sp. at ×100 (**a**) and ×200 (**b**).

The polysaccharide yield was 4.4 (% *w*/*w* dry biomass basis), nearest to the values reported in the diatom *Gomphonema olivaceum* (3% *w*/*w*) [7] and in the benthic seaweed *Sargassum qingdaoense* (7.2% *w*/*w*) [8], but lower than the values reported for the planktonic specie *Spirulina platensis* (13.6% *w*/*w*) [9]. These differences could be due to the extraction methods used and/or the type of species investigated. The extracted polysaccharide consisted of a white-colored powder with fine and granulated parts. Scanning electron microscopy (SEM) can be a useful tool to analyze the surface morphology of polysaccharide powder. The SEM micrographs showed that the lyophilized *Navicula* sp. polysaccharide is an amorphous solid (Figure 1). The observed particles were mostly aggregates of irregular shapes with sharp angles similar to those reported for other sulfated polysaccharides [10].

The main sugars present in the polysaccharide were glucose, galactose, rhamnose, xylose and mannose (Table 1), glucose being the most abundant, with ca. 30% of the polysaccharide dry weight. Staats et al. [11] found that extracellular polysaccharides from *Navicula salinarum* were mainly composed of glucose, galactose, mannose, rhamnose and xylose, with galactose concentrations similar to those found in the present study. On the other hand, Lee et al. [3] reported the presence of fucose, xylose, galactose, mannose and rhamnose in *Navicula directa* extracts, but at higher concentrations (% *w*/*w* dry weight basis) than those found in this study. A small amount of protein (0.48% *w*/*w*) was also detected in the polysaccharide from *Navicula* sp. (Table 1). However, a higher content of protein has been reported for polysaccharides from *Chlorella pyrenoidosa* at different ethanol concentrations (0.75%–11.21% *w*/*w*) [12]. The sulfate content found in the polysaccharide from *Navicula* sp. in this study (0.33%) (Table 1) was in the range reported for a sulfated galactan from the red algae *Ahnfeltia tobuchiensis* (0.2%–0.3% *w*/*w*) [13] but lower than that in other reports for *Navicula* species (8% and 11% *w*/*w*) [3,11]. However, it is well known that the sulfate content in microalgae is highly variable and can range from 0 to approximately 90% [14].

In the present study, the molecular weight (Mw) for the *Navicula* sp. sulfated polysaccharide was 107 kDa, lower than the reported value in another study with *Navicula directa* (222 kDa) [3]. However, it should be mentioned that the information and characterization of sulfated polysaccharides of the genus *Navicula* are still emerging. It should also be noted that the characteristics of microalgae and their biological compounds depend heavily on the culture conditions used and, to an even greater extent, on the species [15,16].

**Figure 2:**
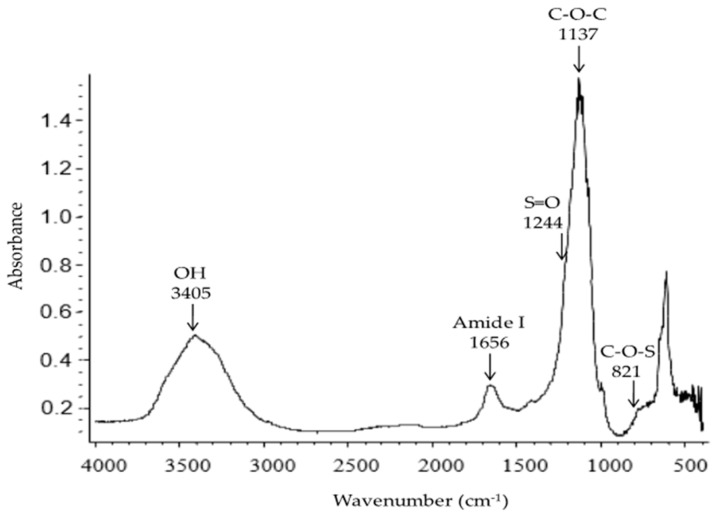
Fourier transform infrared (FT-IR) spectrum of sulfated polysaccharide from *Navicula* sp. The arrows indicate the principal absorption bands.

The Fourier transform infrared (FT*-*IR) spectrum of the sulfated polysaccharide extract showed five distinct bands at wave numbers ranging from 3405–821 cm^−1^ (Figure 2). The bands were assigned to particular functional groups according to previously published literature [17,18]. The spectrum of this polysaccharide showed the typical infrared footprint of carbohydrates. The band in the region of 3405 cm^−1^ corresponds to the stretching vibration characteristic of OH groups; a similar band around this wavenumber was observed for sulfated polysaccharides from green and brown seaweeds [19,20]. The band related to amides associated with the protein was detected at 1656 cm^−1^; this band has also been detected in other microalgae [18]. The most important band was found at 1137 cm^−1^, assigned to C–O–C bending; similar bands were reported for sulfated polysaccharides from brown and red seaweeds [21,22]. The band corresponding to the S=O vibration (1244 cm^−1^) possesses a low intensity; this result could be due to the low sulfate content detected on the sample (0.33% *w*/*w*) as reported in Table 1. Some studies have reported the presence of this band in sulfated polysaccharides extracted from the diatom *Navicula directa* [3] and from the three major groups of seaweeds (green, brown and red algae) [19,20,21,23]. Finally, the band at 821 cm^−1^ was attributed to C–O–S stretching vibrations; sulfated polysaccharides extracted from some green and brown seaweed species also showed a band specific to the C–O–S group [19,20,21,23,24] around a similar wavelength as in our study.

### 2.2. Sulfated Polysaccharide Gelation

**Figure 3:**
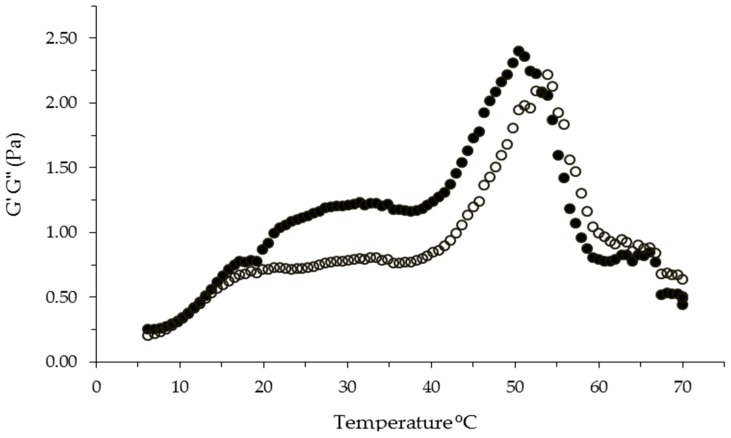
Temperature ramp for sulfated polysaccharide at 1% (*w*/*v*) in the presence of trivalent ions of FeCl_3_ at 0.4% (*w*/*v*) at 1 Hz and 2% strain. G’ (●), G” (○).

Previous experiments were carried out in the present research in order to evaluate the gelation ability of the sulfated polysaccharide from *Navicula* sp. in the presence of mono and divalent cations (KCl and CaCl_2_, respectively). However, for those cations no gelation was observed. When a 0.4% (*w*/*v*) FeCl_3_ solution was dropped into a 1% (*w*/*v*) sulfated polysaccharide aqueous solution, a yellow-orange-colored gel-like substance precipitated as previously reported for λ-carrageenan [6]. The gel-like material was formed after 60 s of FeCl_3_ addition. It has been suggested that trivalent iron metals promote appropriate ionic interactions between sulfated polysaccharide chains, causing their union and subsequent gelation. However, the gelling mechanism of sulfated polysaccharides in the presence of trivalent ions is currently unknown [6,25]. The precipitated coagulum formed in the present study was recovered for further rheological and microstructural characterization. The FeCl_3_-induced gelation of the sulfated polysaccharide from *Navicula* sp. was rheologically investigated by small amplitude oscillatory shear. Figure 3 shows the changes in the elastic (G’) and viscous (G”) moduli of 1% (*w*/*v*) polysaccharide/FeCl_3_ from 5 to 70 °C. The sample showed G’ and G” values of 1.0 and 0.7 Pa, respectively, from 20 to 40 °C, indicating a gelation behavior. Higher G’ and G” values were found in λ-carrageenan/FeCl_3_ gels (G’ = 1200 Pa, G” = 150 Pa) [6] which could be related to a higher sulfate content reported for that sample [26]. In the present study, the polysaccharide/FeCl_3_ gel was thermally stable from 20 to 40 °C, as there was no crossover between G’ and G” in this temperature region. The temperature at which this polysaccharide gel was thermally stable could allow its use in biomedical applications, where the implementation of organic material that supports the body temperature is needed. In a study by Vorvolakos et al. [27], it was observed that hyaluronic acid could form gels in the presence of trivalent cations, a behavior similar to the polysaccharide in our study. The hyaluronic acid gel can be utilized in laparoscopic surgeries to avoid adhesions [28]; because its gelling characteristics were similar to those in our study, *Navicula* sp. sulfated polysaccharide evaluation in that application could be of keen interest.

**Figure 4:**
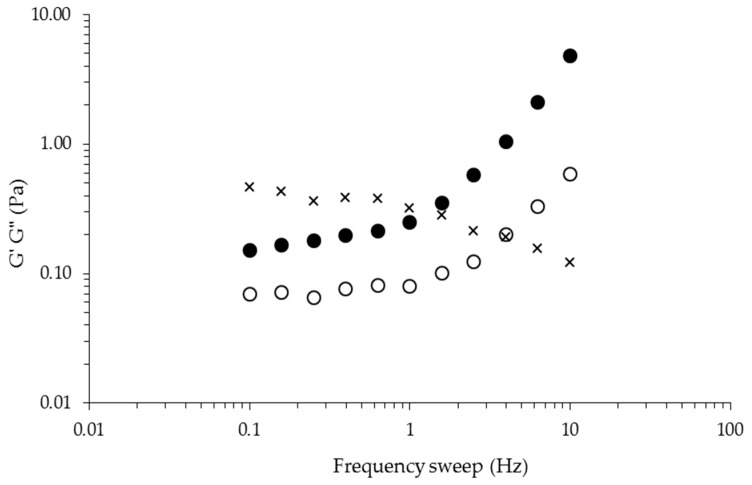
Mechanical spectra of sulfated polysaccharide gel at 1% (*w*/*v*) induced by FeCl_3_ at 0.4% (*w*/*v*). Measurements at 2% strain and 25 °C. G’ (●), G” (○), tan δ (×).

**Figure 5:**
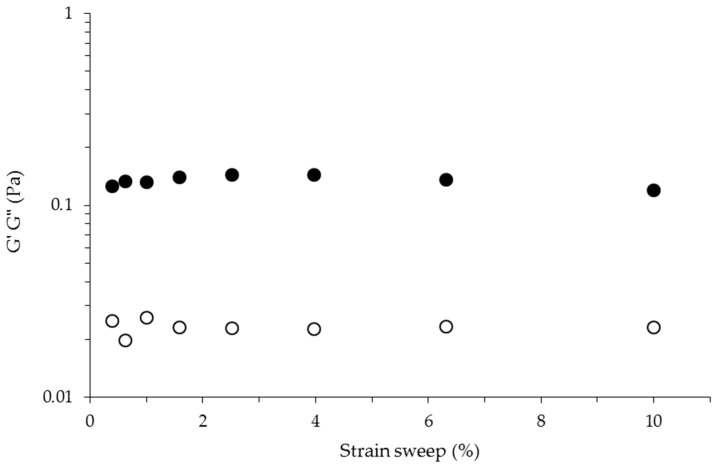
Strain sweep of sulfated polysaccharide gel at 1% (*w*/*v*) induced by FeCl_3_ at 0.4% (*w*/*v*). Measurements at 1 Hz and 25 °C. G’ (●), G” (○).

Mechanical spectra (Figure 4) of the gel were recorded at 25 °C, being typical of a solid-like material with a linear G’ independent of frequency and a G” much smaller than G’ in the frequency interval from 0.1 up to 1.0 Hz [29]. At higher frequency values (from 1.0 up to 10 Hz), G’ and G” enter into the non-linear range as a result of excessive oscillation frequency exposure, corresponding to a weak gel-like behavior [30]. The tangent delta values (tan δ = G”/G’) of the gel as a function of frequency sweep are also presented in Figure 4. Under the experimental conditions used in the present study, the tan δ values registered varied from 0.46 to 0.12 when the frequency changed from 0.1 to 10.0 Hz. These tan δ values are typical of so-called weak gels [29]. When subjected to the strain sweep test, this polysaccharide gel showed a linear behavior from 1.5% to 10.0% strain (Figure 5). The elastic character of this gel could be attributed to the temporary association of sulfated polysaccharide chains during short oscillation periods. It has been suggested that trivalent ions could be more suitable than monovalent ions for balancing the three negative sulfate charges, per disaccharide repeat unit, of polysaccharides such as λ-carrageenan [6].

**Figure 6:**
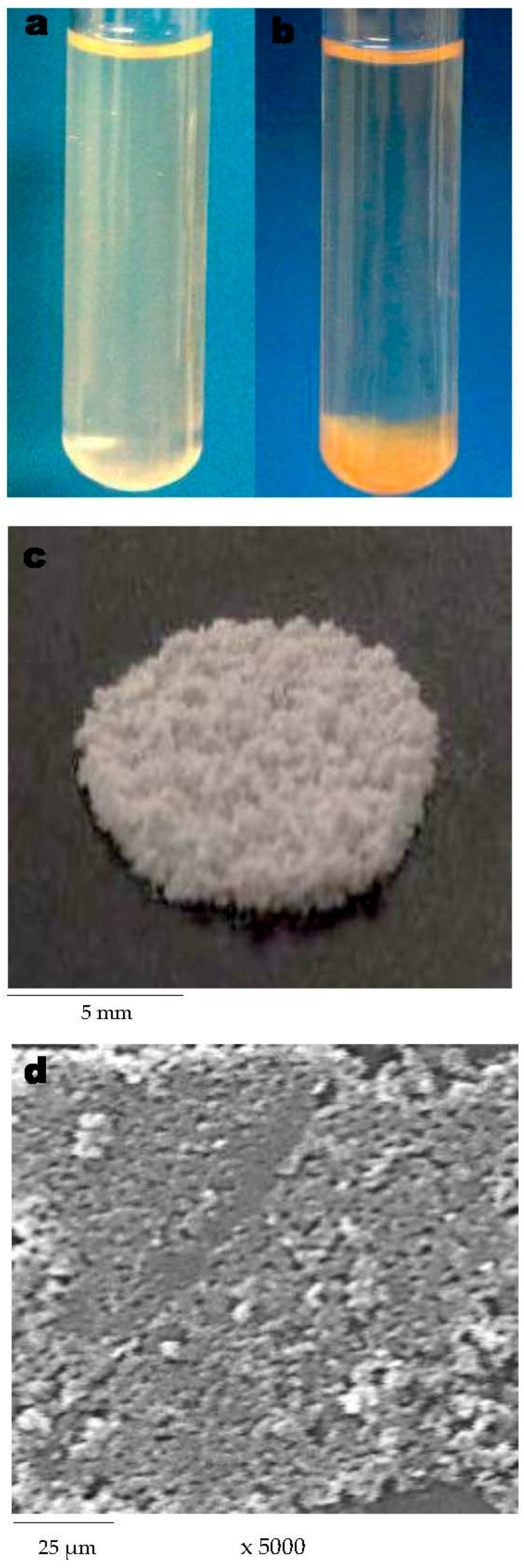
Sulfated polysaccharide from *Navicula* sp. before (**a**) and after (**b**) the addition of FeCl_3_; lyophilized gel (**c**); SEM micrograph of the lyophilized gel (magnification ×5000, scale bar 25 µm) (**d**).

In Figure 6 the sulfated polysaccharide solution before (a) and after (b) FeCl_3_ addition is observed. The yellow gel-like substance was lyophilized (Figure 6c) and analyzed by SEM (Figure 6d). SEM micrographs of the lyophilized polysaccharide gel present a compact pore structure, with an irregular pore size of approximately 150 nm. The gel formed with this trivalent metal consisted of fine-stranded networks with strand thickness on the nm scale. In could be assumed that the SEM preparation method does not affect the sizes of the domains of the network structure. Nevertheless, it is important to note that lyophilized gel does not allow visualizing the original wet-polymeric network but it can be useful to investigate the dried microstructure of the polysaccharide gels.

## 3. Materials and Methods

### 3.1. Materials

The microalgae *Navicula* sp. was obtained and cultured as previously reported [31]. All chemical reagents were purchased from Sigma-Aldrich Chemical Company (St. Louis, MO, USA).

### 3.2. Methods

#### 3.2.1. Extraction of Polysaccharide

At the end of the microalgal culture, the full biomass was harvested by gravity sedimentation method [32] and lyophilized using a Freezone 6 freeze dry system (Labconco, Kansas, MO, USA). Once lyophilized, soluble sulfated polysaccharides were obtained by suspending the lyophilized total biomass in distilled water for 1 h at 30 °C, the suspended biomass was then centrifuged for 15 min at 20,000× *g*. Finally, the supernatant was separated and precipitated overnight under cold conditions with 96% (*v*/*v*) ethanol to allow for the precipitation of sulfated polysaccharides from *Navicula* sp. [11]. Precipitate was recovered and dried by solvent exchange (96% (*v*/*v*) ethanol and pure acetone) and the polysaccharide from *Navicula* sp. was obtained as reported for other marine sulfated polysaccharides [8,9].

#### 3.2.2. Chemical Analysis

The sulfate content of the extracted polysaccharide was determined after hydrolysis with 1 N HCl at 100 °C for 1 h following the sodium-rhodizonate method proposed by Terho and Hartiala [33]. Na_2_SO_4_ was utilized as a standard. The protein content was analyzed using the Dumas method (Leco FP-528 nitrogen analyzer, St. Joseph, MI, USA) [34].

The monosaccharide content was analyzed by gas chromatography (Agilent HP 6890 GC Series, Santa Clara, CA, USA) [35]. Briefly, the polysaccharide sample was hydrolyzed with 3 N H_2_SO_4_ (98% *v*/*v*) at 100 °C, and inositol was added as the internal standard. The external standards were glucose, mannose, galactose, xylose and rhamnose (1 mg/mL, *w*/*v*), which were purchased from Sigma-Aldrich Chemical Company (St. Louis, MO, USA). Sugars were reduced to alditols with sodium borohydride, acetylated with acetic anhydride in the presence of methyl imidazole, and finally extracted with chloroform. After extraction, the alditol-acetates were injected (5 µL) in a DB 225 type column (50% cyanopropylphenyl-dimethylpolysiloxane, 30 m × 0.32 mm ID, 0.15 μm). The gas chromatography conditions were as follows: injection temperature 220 °C, detector temperature 260 °C, and oven temperature programmed to 205 °C at 10 °C/min. Nitrogen was used as the carrier gas and maintained at 1.0 mL/min.

#### 3.2.3. Fourier Transform Infrared (FT-IR) Spectroscopy

The polysaccharide powder and the lyophilized trivalent gels were pressed into KBr pellets. A blank KBr disk was used as background. FT-IR spectrum was recorded on a Nicolet FT-IR spectrophotometer (Nicolet Instruments Corp., Madison, WI, USA). The FT-IR spectrum was measured in absorbance mode from 4000–400 cm^−1^.

#### 3.2.4. Molecular Weight Determination

The molecular characteristics based on the absolute weight-average molecular weight (*M*_W_) of polysaccharide was analyzed by high-performance size-exclusion chromatography (HPSEC) attached to a multiangle laser-light scattering (MALLS) and refractive index (RI) detector (mini-Dawn^®^, Wyatt, Milford, MA, USA). The polysaccharide extract (1 mg/mL *w*/*v*) was dissolved in 100 mM NaNO_3_, filtered through a 0.2 µm membrane, and injected at 25 °C. The RI increment (*dn*/*dc*) utilized for the polysaccharide extract was 0.147 mL/g.

#### 3.2.5. Rheological Measurements

The gelation of the polysaccharide extract was carried out with the following reaction mixture: 1% *w*/*v* of polysaccharide solution with 0.4% *w*/*v* FeCl_3_ in water. For rheological tests, the sulfated polysaccharide gel formation was followed using a strain controller rheometer (Discovery HR-2 rheometer; TA Instruments, New Castle, DE, USA) along with a parallel plate geometry with a plate diameter of 40 mm. A temperature ramp was carried out from 5 to 70 °C at a frequency of 1 Hz and 2% strain. Frequency sweep test was performed from 0.1 to 10 Hz at 2% strain and 25 °C. Strain sweep experiment was done from 0.4 to 10% strain at a 1 Hz frequency and 25 °C. All measurements were performed in duplicate.

#### 3.2.6. Scanning Electron Microscopy Imaging

The polysaccharide powder and the lyophilized gel were all analyzed by field emission scanning electron microscopy (SEM) (JEOL 5410LV, JEOL, Peabody, MA, USA) using a voltage of 10 kV and ×100, ×200 or ×5000 magnifications. SEM images were obtained in secondary and backscattered electrons imaging modes.

## 4. Conclusions

The present study demonstrated that the sulfated polysaccharide from *Navicula* sp. can form gels in the presence of trivalent iron cations and showed the basic viscoelastic and microstructural characteristics of this material. This finding has the potential to expand the utility of sulfated polysaccharides from microalgae in different biotechnological applications and provides a basis for further structural analysis and evaluation of the bioactivities of this sulfated polysaccharide and its trivalent gel.

## Figures and Tables

**Table 1:** Composition of sulfated polysaccharides from *Navicula* sp.

Compounds	% *w*/*w* Dry Weight Basis
Glucose	29.23 ± 2.04
Galactose	21.37 ± 2.27
Rhamnose	10.67 ± 2.66
Xylose	5.18 ± 1.09
Mannose	4.43 ± 0.79
Protein	0.480 ± 0.001
Sulfate	0.330 ± 0.004

All results were obtained from duplicates.
